# (2-Amino­benzoato-κ^2^ *O*,*O*′)(*rac*-5,5,7,12,12,14-hexa­methyl-1,4,8,11-tetra­aza­cyclo­tetra­decane-κ^4^ *N*,*N*′,*N*′′,*N*′′′)nickel(II) perchlorate monohydrate

**DOI:** 10.1107/S1600536810037116

**Published:** 2010-09-25

**Authors:** Guang-Chuan Ou, Seik Weng Ng

**Affiliations:** a Department of Biology and Chemistry, Hunan University of Science and Engineering, Yongzhou Hunan 425100, People’s Republic of China; b Department of Chemistry, University of Malaya, 50603 Kuala Lumpur, Malaysia

## Abstract

In the title salt, [Ni(C_7_H_6_NO_2_)(C_16_H_36_N_4_)]ClO_4_·H_2_O, the Ni^II^ cation is *O*,*O*′-chelated by the benzoate anion and *N*,*N*′,*N*′′,*N*′′′-chelated by the macrocycle ligand, confering a distorted octa­hedral geometry on the metal atom. The complex cations, perchlorate anions and uncoordinated water mol­ecules are linked by N—H⋯O and O—H⋯O hydrogen bonds into a three-dimensional network. The perchlorate ion is disordered over two positions in a 0.554 (8):0.446 (8) ratio.

## Related literature

For two related structures, see: Ou *et al.* (2008*a* ▶,*b* ▶).
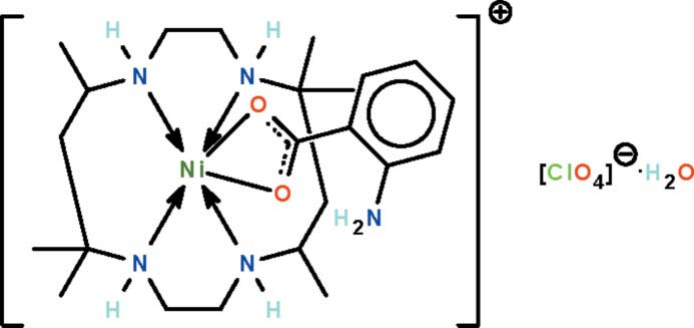


## Experimental

### 

#### Crystal data

[Ni(C_7_H_6_NO_2_)(C_16_H_36_N_4_)]ClO_4_·H_2_O
*M* *_r_* = 596.79
Monoclinic, 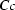

*a* = 9.6452 (5) Å
*b* = 21.5350 (11) Å
*c* = 13.5083 (7) Å
β = 90.784 (1)°
*V* = 2805.5 (3) Å^3^
*Z* = 4
Mo *K*α radiation
μ = 0.84 mm^−1^
*T* = 110 K
0.45 × 0.20 × 0.10 mm

#### Data collection

Bruker SMART APEX diffractometer
Absorption correction: multi-scan (*SADABS*; Sheldrick, 1996 ▶) *T*_min_ = 0.818, *T*_max_ = 1.000
6638 measured reflections
3832 independent reflections
3667 reflections with *I* > 2σ(*I*)
*R*_int_ = 0.016

#### Refinement

*R*[*F* ^2^ > 2σ(*F* ^2^)] = 0.023
*wR*(*F* ^2^) = 0.064
*S* = 1.08
3832 reflections
410 parameters
142 restraints
H atoms treated by a mixture of independent and constrained refinement
Δρ_max_ = 0.25 e Å^−3^
Δρ_min_ = −0.21 e Å^−3^
Absolute structure: Flack (1983 ▶), 874 Friedel pairs
Flack parameter: −0.012 (9)

### 

Data collection: *SMART* (Bruker, 2003 ▶); cell refinement: *SAINT* (Bruker, 2003 ▶); data reduction: *SAINT*; program(s) used to solve structure: *SHELXS97* (Sheldrick, 2008 ▶); program(s) used to refine structure: *SHELXL97* (Sheldrick, 2008 ▶); molecular graphics: *X-SEED* (Barbour, 2001 ▶); software used to prepare material for publication: *publCIF* (Westrip, 2010 ▶).

## Supplementary Material

Crystal structure: contains datablocks global, I. DOI: 10.1107/S1600536810037116/xu5028sup1.cif

Structure factors: contains datablocks I. DOI: 10.1107/S1600536810037116/xu5028Isup2.hkl

Additional supplementary materials:  crystallographic information; 3D view; checkCIF report

## Figures and Tables

**Table 1:** Selected bond lengths (Å)

Ni1—N1	2.124 (2)
Ni1—N2	2.084 (2)
Ni1—N3	2.138 (2)
Ni1—N4	2.087 (2)
Ni1—O1	2.1659 (17)
Ni1—O2	2.1280 (16)

**Table 2:** Hydrogen-bond geometry (Å, °)

*D*—H⋯*A*	*D*—H	H⋯*A*	*D*⋯*A*	*D*—H⋯*A*
N1—H1⋯O1w^i^	0.86 (2)	2.15 (2)	2.995 (3)	166 (2)
N2—H2⋯O5′^ii^	0.86 (2)	2.35 (2)	3.125 (11)	150 (3)
N3—H3⋯O1	0.86 (2)	2.49 (3)	2.885 (3)	109 (2)
N4—H4⋯O6′^ii^	0.86 (3)	2.40 (2)	3.195 (6)	155 (3)
N5—H51⋯O2	0.85 (3)	2.13 (3)	2.751 (3)	129 (2)
N5—H52⋯O4′^i^	0.85 (1)	2.32 (1)	3.155 (6)	168 (3)
O1w—H11⋯O1	0.84 (2)	1.96 (2)	2.795 (2)	172 (3)
O1w—H12⋯O3	0.83 (2)	2.26 (2)	3.077 (9)	167 (3)
O1w—H12⋯O3′	0.83 (2)	2.09 (2)	2.890 (9)	160 (3)

Symmetry codes: (i) 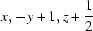; (ii) 
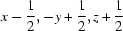
.

**Fig. 1.**
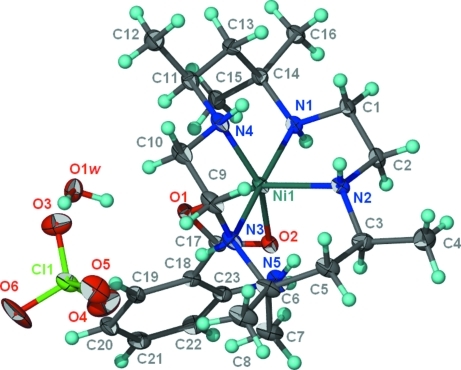
Thermal ellipsoid plot (Barbour, 2001) of [Ni(C16H36N4)(C7H6NO2)][ClO4].H2O at the 50% probability level; hydrogen atoms are shown as spheres of arbitrary radius. The disorder in the perchlorate is not shown.

## supplementary crystallographic information

### Comment

Previous studies on (5,5,7,12,12,14-hexamethyl-1,4,8,11-tetraazacyclotetradecane)nickel benzoate perchlorate salts have documented a chelating mode for the carboxylate ion. The perchlorate counterion is not directly involved in coordination but forms hydrogen bonds with the amino portions of the macrocycle (Ou *et al.*, 2008*a*, 2008*b*). The title substituted benzoate has an additional amino unit that also engages in hydrogen bonding. In the salt, [Ni(C_16_H_36_N_4_)(C_7_H_6_NO_2_)][ClO_4_]^.^H_2_O (Scheme I, Fig. 1), the metal atom is *O*,*O*'-chelated by the carboxylate anion and *N*,*N*',*N*'',*N*'''-chelated by the macrocycle to confer an octahedral geometry to the metal center. The cation, anion and lattice water molecules are linked by N–H···O and O–H···O hydrogen bonds into a three-dimensional network.

### Experimental

Anthranilic acid (0.27 g, 2 mmol) and sodium hydroxide (0.08 g, 2 mmol) were dissolved in water (15 ml). To this solution was added (rac-5,5,7,12,12,14-hexamethyl-1,4,8,11-tetraazacyclotetradecane)nickel diperchlorate (0.11 g, 2 mmol) dissolved in acetonitrile (10 ml). The solution was set aside for the growth of violet crystals after a few days.

### Refinement

Carbon-bound H-atoms were placed in calculated positions (C–H 0.95–1.00 Å) and were included in the refinement in the riding model approximation, with *U*(H) set to 1.2–1.5*U*(C).

The water and amino H atoms were located in a difference Fourier map, and were refined with distance restraints of O–H 0.84±0.01 Å and N–H 0.86±0.01 Å. For the water molecule, the H···H distance was restrained to 1.37±0.01 Å. The temperature factors were tied to those of the parent atoms by a factor of 1.2–1.5 times.

The perchlorate is disordered over two positions in a 0.554 (8):0.446 (8) ratio. All Cl–O distances were restrained to within 0.01 Å of each other as were the O···O distances. The anisotropic temperature factors of the O atoms were restrained to be nearly isotropic.

### Crystal data

**Table:**

[Ni(C_7_H_6_NO_2_)(C_16_H_36_N_4_)]ClO_4_·H_2_O	*F*(000) = 1272
*M**_r_* = 596.79	*D*_x_ = 1.413 Mg m^−^^3^
Monoclinic, *C**c*	Mo *K*α radiation, λ = 0.71073 Å
Hall symbol: C -2yc	Cell parameters from 5267 reflections
*a* = 9.6452 (5) Å	θ = 2.3–27.0°
*b* = 21.5350 (11) Å	µ = 0.84 mm^−^^1^
*c* = 13.5083 (7) Å	*T* = 110 K
β = 90.784 (1)°	Prism, violet
*V* = 2805.5 (3) Å^3^	0.45 × 0.20 × 0.10 mm
*Z* = 4	

### Data collection

**Table:**

Bruker SMART APEX diffractometer	3832 independent reflections
Radiation source: fine-focus sealed tube	3667 reflections with *I* > 2σ(*I*)
graphite	*R*_int_ = 0.016
φ and ω scans	θ_max_ = 27.0°, θ_min_ = 1.9°
Absorption correction: multi-scan (*SADABS*; Sheldrick, 1996)	*h* = −11→12
*T*_min_ = 0.818, *T*_max_ = 1.000	*k* = −26→26
6638 measured reflections	*l* = −8→17

### Refinement

**Table:**

Refinement on *F*^2^	Secondary atom site location: difference Fourier map
Least-squares matrix: full	Hydrogen site location: inferred from neighbouring sites
*R*[*F*^2^ > 2σ(*F*^2^)] = 0.023	H atoms treated by a mixture of independent and constrained refinement
*wR*(*F*^2^) = 0.064	*w* = 1/[σ^2^(*F*_o_^2^) + (0.0373*P*)^2^ + 0.1275*P*] where *P* = (*F*_o_^2^ + 2*F*_c_^2^)/3
*S* = 1.08	(Δ/σ)_max_ = 0.001
3832 reflections	Δρ_max_ = 0.25 e Å^−^^3^
410 parameters	Δρ_min_ = −0.21 e Å^−^^3^
142 restraints	Absolute structure: Flack (1983), 874 Friedel pairs
Primary atom site location: structure-invariant direct methods	Flack parameter: −0.012 (9)

### Fractional atomic coordinates and isotropic or equivalent isotropic displacement parameters (Å^2^)

**Table:**

	*x*	*y*	*z*	*U*_iso_*/*U*_eq_	Occ. (<1)
Ni1	0.50011 (3)	0.399116 (12)	0.49992 (3)	0.01859 (8)	
O1	0.58175 (16)	0.44546 (8)	0.37089 (12)	0.0219 (4)	
O2	0.66792 (16)	0.46225 (7)	0.51979 (12)	0.0201 (3)	
Cl1	0.6956 (5)	0.2675 (2)	0.1539 (3)	0.0295 (7)	0.554 (8)
O3	0.5575 (5)	0.2886 (4)	0.1347 (6)	0.0444 (19)	0.554 (8)
O4	0.7620 (5)	0.3111 (2)	0.2197 (4)	0.0581 (17)	0.554 (8)
O5	0.6945 (9)	0.2080 (2)	0.2001 (5)	0.0439 (19)	0.554 (8)
O6	0.7708 (7)	0.2650 (3)	0.0641 (4)	0.070 (2)	0.554 (8)
Cl1'	0.6956 (6)	0.2641 (3)	0.1262 (4)	0.0311 (10)	0.446 (8)
O3'	0.5794 (8)	0.2974 (4)	0.1623 (6)	0.042 (2)	0.446 (8)
O4'	0.8213 (6)	0.2957 (3)	0.1496 (6)	0.060 (3)	0.446 (8)
O5'	0.6992 (12)	0.2028 (3)	0.1656 (9)	0.057 (3)	0.446 (8)
O6'	0.6838 (8)	0.2577 (3)	0.0218 (4)	0.058 (2)	0.446 (8)
O1W	0.5173 (2)	0.42855 (9)	0.17034 (14)	0.0296 (4)	
H11	0.545 (3)	0.4343 (13)	0.2291 (11)	0.044*	
H12	0.537 (4)	0.3925 (8)	0.153 (2)	0.044*	
N1	0.3714 (2)	0.46844 (10)	0.56243 (16)	0.0190 (4)	
H1	0.423 (2)	0.4982 (10)	0.585 (2)	0.023*	
N2	0.4909 (2)	0.35763 (9)	0.63905 (16)	0.0211 (4)	
H2	0.428 (2)	0.3296 (10)	0.636 (2)	0.025*	
N3	0.6127 (2)	0.32080 (10)	0.44631 (17)	0.0243 (4)	
H3	0.652 (3)	0.3351 (13)	0.3944 (14)	0.029*	
N4	0.3296 (2)	0.36063 (10)	0.42555 (16)	0.0242 (4)	
H4	0.280 (3)	0.3380 (12)	0.4632 (19)	0.029*	
N5	0.7978 (2)	0.57537 (11)	0.53966 (18)	0.0289 (5)	
H51	0.739 (3)	0.5514 (12)	0.567 (2)	0.035*	
H52	0.814 (3)	0.6115 (8)	0.563 (2)	0.035*	
C1	0.3207 (3)	0.44029 (12)	0.65532 (18)	0.0245 (5)	
H1A	0.2438	0.4112	0.6402	0.029*	
H1B	0.2852	0.4732	0.6995	0.029*	
C2	0.4374 (3)	0.40601 (11)	0.70620 (19)	0.0253 (5)	
H2A	0.5126	0.4354	0.7241	0.030*	
H2B	0.4037	0.3866	0.7678	0.030*	
C3	0.6234 (3)	0.33020 (13)	0.6779 (2)	0.0282 (6)	
H3a	0.6968	0.3629	0.6751	0.034*	
C4	0.6109 (3)	0.30893 (15)	0.7856 (2)	0.0378 (7)	
H4A	0.5814	0.3440	0.8265	0.057*	
H4B	0.7011	0.2937	0.8096	0.057*	
H4C	0.5423	0.2755	0.7895	0.057*	
C5	0.6684 (3)	0.27612 (12)	0.6133 (2)	0.0293 (6)	
H5A	0.5883	0.2477	0.6059	0.035*	
H5B	0.7418	0.2532	0.6499	0.035*	
C6	0.7230 (3)	0.28992 (12)	0.5088 (2)	0.0298 (6)	
C7	0.8464 (3)	0.33447 (13)	0.5146 (2)	0.0353 (7)	
H7A	0.8832	0.3409	0.4481	0.053*	
H7B	0.9188	0.3167	0.5575	0.053*	
H7C	0.8162	0.3743	0.5418	0.053*	
C8	0.7727 (3)	0.22831 (14)	0.4626 (3)	0.0393 (7)	
H8A	0.7921	0.2348	0.3923	0.059*	
H8B	0.7003	0.1967	0.4692	0.059*	
H8C	0.8573	0.2143	0.4969	0.059*	
C9	0.5037 (3)	0.27846 (12)	0.4086 (2)	0.0297 (6)	
H9A	0.4632	0.2551	0.4643	0.036*	
H9B	0.5444	0.2483	0.3620	0.036*	
C10	0.3920 (3)	0.31573 (13)	0.3564 (2)	0.0288 (6)	
H10A	0.4324	0.3383	0.2998	0.035*	
H10B	0.3194	0.2874	0.3304	0.035*	
C11	0.2384 (3)	0.40671 (11)	0.37461 (19)	0.0241 (5)	
H11A	0.2979	0.4328	0.3310	0.029*	
C12	0.1271 (3)	0.37581 (15)	0.3090 (2)	0.0335 (6)	
H12A	0.1719	0.3517	0.2568	0.050*	
H12B	0.0680	0.4078	0.2788	0.050*	
H12C	0.0704	0.3481	0.3494	0.050*	
C13	0.1684 (2)	0.45014 (12)	0.44934 (19)	0.0238 (5)	
H13A	0.1253	0.4240	0.5007	0.029*	
H13B	0.0923	0.4722	0.4142	0.029*	
C14	0.2594 (2)	0.49930 (12)	0.50235 (18)	0.0217 (5)	
C15	0.3297 (3)	0.54169 (12)	0.42814 (19)	0.0237 (5)	
H15A	0.3819	0.5740	0.4637	0.036*	
H15B	0.2592	0.5611	0.3853	0.036*	
H15C	0.3932	0.5173	0.3876	0.036*	
C16	0.1671 (3)	0.54025 (13)	0.5675 (2)	0.0259 (6)	
H16A	0.2256	0.5678	0.6079	0.039*	
H16B	0.1117	0.5138	0.6109	0.039*	
H16C	0.1053	0.5652	0.5253	0.039*	
C17	0.6690 (2)	0.47306 (11)	0.42754 (19)	0.0186 (5)	
C18	0.7701 (2)	0.51743 (11)	0.38477 (18)	0.0198 (5)	
C19	0.8068 (2)	0.51078 (12)	0.28554 (19)	0.0234 (5)	
H19	0.7678	0.4777	0.2479	0.028*	
C20	0.8984 (3)	0.55104 (13)	0.2407 (2)	0.0292 (6)	
H20	0.9247	0.5450	0.1739	0.035*	
C21	0.9514 (3)	0.60087 (12)	0.2959 (2)	0.0301 (6)	
H21	1.0125	0.6296	0.2657	0.036*	
C22	0.9161 (3)	0.60871 (12)	0.3934 (2)	0.0276 (6)	
H22	0.9532	0.6430	0.4292	0.033*	
C23	0.8259 (2)	0.56699 (12)	0.4417 (2)	0.0219 (5)	

### Atomic displacement parameters (Å^2^)

**Table:**

	*U*^11^	*U*^22^	*U*^33^	*U*^12^	*U*^13^	*U*^23^
Ni1	0.01972 (13)	0.01555 (14)	0.02064 (13)	−0.00292 (12)	0.00605 (9)	−0.00109 (13)
O1	0.0225 (8)	0.0217 (9)	0.0217 (8)	−0.0068 (7)	0.0050 (7)	−0.0017 (7)
O2	0.0227 (8)	0.0171 (9)	0.0207 (8)	−0.0015 (6)	0.0043 (7)	−0.0002 (7)
Cl1	0.0273 (9)	0.0245 (10)	0.0371 (17)	0.0031 (6)	0.0107 (11)	0.0046 (11)
O3	0.036 (2)	0.035 (3)	0.062 (4)	0.005 (2)	−0.007 (2)	−0.016 (3)
O4	0.053 (3)	0.045 (3)	0.075 (4)	−0.004 (2)	−0.017 (3)	−0.008 (2)
O5	0.055 (3)	0.015 (2)	0.062 (4)	0.000 (2)	0.014 (3)	0.010 (2)
O6	0.071 (4)	0.077 (4)	0.065 (4)	0.031 (3)	0.052 (4)	0.012 (3)
Cl1'	0.0308 (12)	0.0213 (12)	0.041 (3)	0.0025 (8)	0.0045 (16)	0.0042 (15)
O3'	0.048 (4)	0.033 (4)	0.047 (4)	0.016 (3)	0.024 (3)	0.001 (3)
O4'	0.055 (3)	0.038 (3)	0.087 (6)	−0.014 (3)	−0.021 (3)	0.020 (3)
O5'	0.051 (4)	0.027 (4)	0.094 (7)	0.008 (3)	0.017 (5)	0.010 (4)
O6'	0.055 (4)	0.079 (4)	0.041 (3)	0.016 (3)	0.009 (3)	−0.017 (3)
O1W	0.0405 (11)	0.0243 (10)	0.0239 (9)	0.0039 (8)	−0.0026 (8)	0.0000 (7)
N1	0.0222 (10)	0.0145 (10)	0.0205 (10)	−0.0003 (8)	0.0027 (8)	0.0008 (8)
N2	0.0208 (10)	0.0180 (10)	0.0248 (10)	0.0008 (8)	0.0057 (8)	0.0009 (8)
N3	0.0254 (10)	0.0186 (11)	0.0291 (11)	−0.0042 (8)	0.0107 (9)	−0.0014 (9)
N4	0.0258 (10)	0.0228 (11)	0.0242 (10)	−0.0061 (8)	0.0086 (8)	−0.0022 (9)
N5	0.0337 (12)	0.0215 (11)	0.0315 (12)	−0.0095 (9)	0.0063 (9)	−0.0075 (9)
C1	0.0287 (12)	0.0262 (13)	0.0187 (11)	0.0058 (10)	0.0078 (10)	0.0013 (10)
C2	0.0314 (13)	0.0246 (13)	0.0202 (11)	0.0062 (10)	0.0056 (10)	0.0018 (10)
C3	0.0250 (12)	0.0240 (14)	0.0356 (15)	0.0034 (10)	0.0030 (11)	0.0032 (11)
C4	0.0416 (16)	0.0366 (16)	0.0353 (15)	0.0169 (13)	0.0014 (13)	0.0070 (13)
C5	0.0295 (13)	0.0180 (12)	0.0406 (15)	0.0043 (10)	0.0081 (11)	0.0038 (11)
C6	0.0245 (13)	0.0232 (14)	0.0420 (16)	0.0016 (10)	0.0122 (11)	−0.0024 (12)
C7	0.0229 (13)	0.0326 (16)	0.0507 (17)	0.0009 (11)	0.0090 (12)	−0.0002 (13)
C8	0.0370 (16)	0.0235 (15)	0.058 (2)	0.0074 (12)	0.0127 (15)	−0.0063 (14)
C9	0.0347 (13)	0.0181 (13)	0.0368 (14)	−0.0057 (10)	0.0146 (11)	−0.0073 (11)
C10	0.0315 (14)	0.0250 (14)	0.0301 (14)	−0.0081 (10)	0.0079 (11)	−0.0125 (11)
C11	0.0247 (12)	0.0247 (13)	0.0232 (12)	−0.0063 (9)	0.0049 (10)	−0.0010 (10)
C12	0.0295 (13)	0.0429 (17)	0.0282 (13)	−0.0081 (12)	0.0010 (11)	−0.0065 (12)
C13	0.0193 (11)	0.0276 (13)	0.0245 (12)	−0.0013 (9)	0.0031 (10)	0.0017 (10)
C14	0.0229 (11)	0.0224 (12)	0.0200 (11)	0.0015 (9)	0.0040 (9)	0.0031 (10)
C15	0.0278 (12)	0.0222 (12)	0.0212 (11)	0.0021 (9)	0.0048 (10)	0.0034 (10)
C16	0.0263 (13)	0.0259 (15)	0.0256 (13)	0.0059 (10)	0.0062 (10)	0.0040 (11)
C17	0.0197 (11)	0.0140 (11)	0.0222 (12)	0.0024 (9)	0.0045 (9)	−0.0008 (9)
C18	0.0190 (11)	0.0170 (12)	0.0236 (12)	0.0000 (8)	0.0017 (9)	0.0014 (9)
C19	0.0210 (12)	0.0238 (13)	0.0255 (12)	−0.0011 (9)	0.0037 (10)	0.0014 (10)
C20	0.0253 (12)	0.0355 (15)	0.0269 (13)	0.0006 (10)	0.0083 (10)	0.0080 (11)
C21	0.0210 (12)	0.0288 (14)	0.0405 (15)	−0.0034 (9)	0.0045 (11)	0.0131 (12)
C22	0.0249 (12)	0.0199 (13)	0.0378 (15)	−0.0048 (9)	−0.0005 (11)	0.0034 (11)
C23	0.0194 (11)	0.0193 (12)	0.0269 (12)	0.0015 (9)	0.0022 (10)	0.0017 (10)

### Geometric parameters (Å, °)

**Table:**

Ni1—N1	2.124 (2)	C5—C6	1.541 (4)
Ni1—N2	2.084 (2)	C5—H5A	0.9900
Ni1—N3	2.138 (2)	C5—H5B	0.9900
Ni1—N4	2.087 (2)	C6—C7	1.530 (4)
Ni1—O1	2.1659 (17)	C6—C8	1.546 (4)
Ni1—O2	2.1280 (16)	C7—H7A	0.9800
O1—C17	1.276 (3)	C7—H7B	0.9800
O2—C17	1.268 (3)	C7—H7C	0.9800
Cl1—O6	1.423 (4)	C8—H8A	0.9800
Cl1—O5	1.425 (5)	C8—H8B	0.9800
Cl1—O3	1.427 (5)	C8—H8C	0.9800
Cl1—O4	1.437 (5)	C9—C10	1.510 (4)
Cl1'—O6'	1.421 (5)	C9—H9A	0.9900
Cl1'—O5'	1.423 (6)	C9—H9B	0.9900
Cl1'—O3'	1.422 (5)	C10—H10A	0.9900
Cl1'—O4'	1.422 (6)	C10—H10B	0.9900
O1W—H11	0.843 (10)	C11—C12	1.534 (4)
O1W—H12	0.833 (10)	C11—C13	1.539 (3)
N1—C1	1.482 (3)	C11—H11A	1.0000
N1—C14	1.498 (3)	C12—H12A	0.9800
N1—H1	0.861 (10)	C12—H12B	0.9800
N2—C2	1.479 (3)	C12—H12C	0.9800
N2—C3	1.496 (3)	C13—C14	1.545 (3)
N2—H2	0.858 (10)	C13—H13A	0.9900
N3—C9	1.477 (3)	C13—H13B	0.9900
N3—C6	1.504 (4)	C14—C15	1.522 (3)
N3—H3	0.860 (10)	C14—C16	1.538 (3)
N4—C10	1.478 (3)	C15—H15A	0.9800
N4—C11	1.489 (3)	C15—H15B	0.9800
N4—H4	0.86 (3)	C15—H15C	0.9800
N5—C23	1.366 (3)	C16—H16A	0.9800
N5—H51	0.86 (3)	C16—H16B	0.9800
N5—H52	0.854 (10)	C16—H16C	0.9800
C1—C2	1.504 (3)	C17—C18	1.487 (3)
C1—H1A	0.9900	C18—C19	1.399 (3)
C1—H1B	0.9900	C18—C23	1.418 (3)
C2—H2A	0.9900	C19—C20	1.383 (3)
C2—H2B	0.9900	C19—H19	0.9500
C3—C5	1.522 (4)	C20—C21	1.400 (4)
C3—C4	1.532 (4)	C20—H20	0.9500
C3—H3a	1.0000	C21—C22	1.375 (4)
C4—H4A	0.9800	C21—H21	0.9500
C4—H4B	0.9800	C22—C23	1.416 (4)
C4—H4C	0.9800	C22—H22	0.9500
			
N2—Ni1—N4	102.76 (8)	N3—C6—C7	107.2 (2)
N2—Ni1—N1	84.86 (8)	N3—C6—C5	110.60 (19)
N4—Ni1—N1	90.54 (8)	C7—C6—C5	110.5 (2)
N2—Ni1—O2	101.64 (7)	N3—C6—C8	111.9 (2)
N4—Ni1—O2	155.16 (8)	C7—C6—C8	108.2 (2)
N1—Ni1—O2	87.05 (7)	C5—C6—C8	108.4 (2)
N2—Ni1—N3	89.73 (8)	C6—C7—H7A	109.5
N4—Ni1—N3	85.66 (8)	C6—C7—H7B	109.5
N1—Ni1—N3	172.56 (8)	H7A—C7—H7B	109.5
O2—Ni1—N3	99.07 (7)	C6—C7—H7C	109.5
N2—Ni1—O1	160.67 (7)	H7A—C7—H7C	109.5
N4—Ni1—O1	95.07 (7)	H7B—C7—H7C	109.5
N1—Ni1—O1	102.53 (7)	C6—C8—H8A	109.5
O2—Ni1—O1	61.50 (6)	C6—C8—H8B	109.5
N3—Ni1—O1	84.20 (7)	H8A—C8—H8B	109.5
C17—O1—Ni1	88.63 (14)	C6—C8—H8C	109.5
C17—O2—Ni1	90.56 (14)	H8A—C8—H8C	109.5
O6—Cl1—O5	110.2 (4)	H8B—C8—H8C	109.5
O6—Cl1—O3	110.1 (4)	N3—C9—C10	109.4 (2)
O5—Cl1—O3	110.7 (4)	N3—C9—H9A	109.8
O6—Cl1—O4	108.9 (4)	C10—C9—H9A	109.8
O5—Cl1—O4	108.8 (4)	N3—C9—H9B	109.8
O3—Cl1—O4	108.1 (4)	C10—C9—H9B	109.8
O6'—Cl1'—O5'	106.4 (7)	H9A—C9—H9B	108.2
O6'—Cl1'—O3'	109.6 (5)	N4—C10—C9	110.3 (2)
O5'—Cl1'—O3'	110.8 (5)	N4—C10—H10A	109.6
O6'—Cl1'—O4'	108.9 (5)	C9—C10—H10A	109.6
O5'—Cl1'—O4'	110.2 (5)	N4—C10—H10B	109.6
O3'—Cl1'—O4'	110.8 (5)	C9—C10—H10B	109.6
H11—O1W—H12	109.4 (17)	H10A—C10—H10B	108.1
C1—N1—C14	113.35 (19)	N4—C11—C12	112.5 (2)
C1—N1—Ni1	104.50 (15)	N4—C11—C13	111.3 (2)
C14—N1—Ni1	121.17 (16)	C12—C11—C13	109.5 (2)
C1—N1—H1	101.7 (19)	N4—C11—H11A	107.8
C14—N1—H1	105.3 (19)	C12—C11—H11A	107.8
Ni1—N1—H1	109.1 (18)	C13—C11—H11A	107.8
C2—N2—C3	111.5 (2)	C11—C12—H12A	109.5
C2—N2—Ni1	105.70 (15)	C11—C12—H12B	109.5
C3—N2—Ni1	116.01 (15)	H12A—C12—H12B	109.5
C2—N2—H2	106 (2)	C11—C12—H12C	109.5
C3—N2—H2	110 (2)	H12A—C12—H12C	109.5
Ni1—N2—H2	107 (2)	H12B—C12—H12C	109.5
C9—N3—C6	114.5 (2)	C11—C13—C14	117.97 (19)
C9—N3—Ni1	103.96 (15)	C11—C13—H13A	107.8
C6—N3—Ni1	121.14 (16)	C14—C13—H13A	107.8
C9—N3—H3	105 (2)	C11—C13—H13B	107.8
C6—N3—H3	107 (2)	C14—C13—H13B	107.8
Ni1—N3—H3	103 (2)	H13A—C13—H13B	107.2
C10—N4—C11	112.8 (2)	N1—C14—C15	107.37 (19)
C10—N4—Ni1	103.81 (15)	N1—C14—C16	111.3 (2)
C11—N4—Ni1	114.50 (15)	C15—C14—C16	107.4 (2)
C10—N4—H4	104 (2)	N1—C14—C13	110.4 (2)
C11—N4—H4	109 (2)	C15—C14—C13	111.2 (2)
Ni1—N4—H4	113 (2)	C16—C14—C13	109.19 (19)
C23—N5—H51	119 (2)	C14—C15—H15A	109.5
C23—N5—H52	116 (2)	C14—C15—H15B	109.5
H51—N5—H52	121 (3)	H15A—C15—H15B	109.5
N1—C1—C2	109.5 (2)	C14—C15—H15C	109.5
N1—C1—H1A	109.8	H15A—C15—H15C	109.5
C2—C1—H1A	109.8	H15B—C15—H15C	109.5
N1—C1—H1B	109.8	C14—C16—H16A	109.5
C2—C1—H1B	109.8	C14—C16—H16B	109.5
H1A—C1—H1B	108.2	H16A—C16—H16B	109.5
N2—C2—C1	109.3 (2)	C14—C16—H16C	109.5
N2—C2—H2A	109.8	H16A—C16—H16C	109.5
C1—C2—H2A	109.8	H16B—C16—H16C	109.5
N2—C2—H2B	109.8	O2—C17—O1	119.3 (2)
C1—C2—H2B	109.8	O2—C17—C18	120.9 (2)
H2A—C2—H2B	108.3	O1—C17—C18	119.8 (2)
N2—C3—C5	110.5 (2)	C19—C18—C23	119.9 (2)
N2—C3—C4	111.9 (2)	C19—C18—C17	118.9 (2)
C5—C3—C4	110.0 (2)	C23—C18—C17	121.3 (2)
N2—C3—H3a	108.1	C20—C19—C18	121.8 (2)
C5—C3—H3a	108.1	C20—C19—H19	119.1
C4—C3—H3a	108.1	C18—C19—H19	119.1
C3—C4—H4A	109.5	C19—C20—C21	118.6 (2)
C3—C4—H4B	109.5	C19—C20—H20	120.7
H4A—C4—H4B	109.5	C21—C20—H20	120.7
C3—C4—H4C	109.5	C22—C21—C20	120.7 (2)
H4A—C4—H4C	109.5	C22—C21—H21	119.6
H4B—C4—H4C	109.5	C20—C21—H21	119.6
C3—C5—C6	118.7 (2)	C21—C22—C23	121.7 (3)
C3—C5—H5A	107.6	C21—C22—H22	119.1
C6—C5—H5A	107.6	C23—C22—H22	119.1
C3—C5—H5B	107.6	N5—C23—C18	123.1 (2)
C6—C5—H5B	107.6	N5—C23—C22	119.6 (2)
H5A—C5—H5B	107.1	C18—C23—C22	117.3 (2)
			
N2—Ni1—O1—C17	−31.3 (3)	C2—N2—C3—C4	51.2 (3)
N4—Ni1—O1—C17	171.28 (14)	Ni1—N2—C3—C4	172.23 (19)
N1—Ni1—O1—C17	79.59 (14)	N2—C3—C5—C6	71.2 (3)
O2—Ni1—O1—C17	0.00 (13)	C4—C3—C5—C6	−164.7 (2)
N3—Ni1—O1—C17	−103.62 (14)	C9—N3—C6—C7	163.1 (2)
N2—Ni1—O2—C17	169.89 (14)	Ni1—N3—C6—C7	−71.0 (2)
N4—Ni1—O2—C17	−21.1 (2)	C9—N3—C6—C5	−76.4 (3)
N1—Ni1—O2—C17	−105.97 (14)	Ni1—N3—C6—C5	49.4 (3)
N3—Ni1—O2—C17	78.28 (15)	C9—N3—C6—C8	44.6 (3)
O1—Ni1—O2—C17	0.00 (13)	Ni1—N3—C6—C8	170.46 (18)
N2—Ni1—N1—C1	−14.19 (16)	C3—C5—C6—N3	−61.6 (3)
N4—Ni1—N1—C1	88.57 (16)	C3—C5—C6—C7	56.8 (3)
O2—Ni1—N1—C1	−116.17 (16)	C3—C5—C6—C8	175.3 (2)
O1—Ni1—N1—C1	−176.11 (15)	C6—N3—C9—C10	174.1 (2)
N2—Ni1—N1—C14	−143.61 (18)	Ni1—N3—C9—C10	39.7 (2)
N4—Ni1—N1—C14	−40.84 (18)	C11—N4—C10—C9	169.1 (2)
O2—Ni1—N1—C14	114.42 (17)	Ni1—N4—C10—C9	44.6 (2)
O1—Ni1—N1—C14	54.48 (18)	N3—C9—C10—N4	−59.9 (3)
N4—Ni1—N2—C2	−104.54 (16)	C10—N4—C11—C12	53.3 (3)
N1—Ni1—N2—C2	−15.15 (16)	Ni1—N4—C11—C12	171.73 (16)
O2—Ni1—N2—C2	70.77 (16)	C10—N4—C11—C13	176.57 (19)
N3—Ni1—N2—C2	169.98 (16)	Ni1—N4—C11—C13	−65.0 (2)
O1—Ni1—N2—C2	98.6 (2)	N4—C11—C13—C14	71.8 (3)
N4—Ni1—N2—C3	131.31 (17)	C12—C11—C13—C14	−163.2 (2)
N1—Ni1—N2—C3	−139.30 (18)	C1—N1—C14—C15	163.3 (2)
O2—Ni1—N2—C3	−53.38 (18)	Ni1—N1—C14—C15	−71.2 (2)
N3—Ni1—N2—C3	45.83 (18)	C1—N1—C14—C16	46.1 (3)
O1—Ni1—N2—C3	−25.6 (3)	Ni1—N1—C14—C16	171.49 (16)
N2—Ni1—N3—C9	90.51 (16)	C1—N1—C14—C13	−75.4 (2)
N4—Ni1—N3—C9	−12.31 (16)	Ni1—N1—C14—C13	50.1 (2)
O2—Ni1—N3—C9	−167.74 (16)	C11—C13—C14—N1	−61.3 (3)
O1—Ni1—N3—C9	−107.87 (16)	C11—C13—C14—C15	57.7 (3)
N2—Ni1—N3—C6	−40.04 (18)	C11—C13—C14—C16	176.0 (2)
N4—Ni1—N3—C6	−142.86 (18)	Ni1—O2—C17—O1	0.0 (2)
O2—Ni1—N3—C6	61.70 (18)	Ni1—O2—C17—C18	179.87 (19)
O1—Ni1—N3—C6	121.58 (18)	Ni1—O1—C17—O2	0.0 (2)
N2—Ni1—N4—C10	−105.88 (17)	Ni1—O1—C17—C18	−179.88 (19)
N1—Ni1—N4—C10	169.26 (17)	O2—C17—C18—C19	155.0 (2)
O2—Ni1—N4—C10	85.1 (2)	O1—C17—C18—C19	−25.2 (3)
N3—Ni1—N4—C10	−17.14 (17)	O2—C17—C18—C23	−26.9 (3)
O1—Ni1—N4—C10	66.63 (17)	O1—C17—C18—C23	152.9 (2)
N2—Ni1—N4—C11	130.71 (15)	C23—C18—C19—C20	0.8 (4)
N1—Ni1—N4—C11	45.86 (16)	C17—C18—C19—C20	178.9 (2)
O2—Ni1—N4—C11	−38.3 (3)	C18—C19—C20—C21	−2.2 (4)
N3—Ni1—N4—C11	−140.55 (16)	C19—C20—C21—C22	1.7 (4)
O1—Ni1—N4—C11	−56.77 (16)	C20—C21—C22—C23	0.3 (4)
C14—N1—C1—C2	175.3 (2)	C19—C18—C23—N5	−178.1 (2)
Ni1—N1—C1—C2	41.4 (2)	C17—C18—C23—N5	3.8 (4)
C3—N2—C2—C1	169.3 (2)	C19—C18—C23—C22	1.1 (3)
Ni1—N2—C2—C1	42.4 (2)	C17—C18—C23—C22	−176.9 (2)
N1—C1—C2—N2	−58.6 (3)	C21—C22—C23—N5	177.6 (2)
C2—N2—C3—C5	174.1 (2)	C21—C22—C23—C18	−1.7 (4)
Ni1—N2—C3—C5	−64.8 (2)		

### Hydrogen-bond geometry (Å, °)

**Table:**

*D*—H···*A*	*D*—H	H···*A*	*D*···*A*	*D*—H···*A*
N1—H1···O1w^i^	0.86 (2)	2.15 (2)	2.995 (3)	166 (2)
N2—H2···O5^ii^	0.86 (2)	2.56 (2)	3.304 (8)	147 (2)
N2—H2···O5'^ii^	0.86 (2)	2.35 (2)	3.125 (11)	150 (3)
N3—H3···O1	0.86 (2)	2.49 (3)	2.885 (3)	109 (2)
N4—H4···O6^ii^	0.86 (3)	2.61 (2)	3.343 (6)	145 (3)
N4—H4···O6'^ii^	0.86 (3)	2.40 (2)	3.195 (6)	155 (3)
N5—H51···O2	0.85 (3)	2.13 (3)	2.751 (3)	129 (2)
N5—H52···O4'^i^	0.85 (1)	2.32 (1)	3.155 (6)	168 (3)
O1w—H11···O1	0.84 (2)	1.96 (2)	2.795 (2)	172 (3)
O1w—H12···O3	0.83 (2)	2.26 (2)	3.077 (9)	167 (3)
O1w—H12···O3'	0.83 (2)	2.09 (2)	2.890 (9)	160 (3)

Symmetry codes: (i) *x*, −*y*+1, *z*+1/2; (ii) *x*−1/2, −*y*+1/2, *z*+1/2.

## References

[bb1] Barbour, L. J. (2001). *J. Supramol. Chem.* **1**, 189–191.

[bb2] Bruker (2003). *SAINT* and *SMART* Bruker AXS Inc., Madison, Wisconsin, USA.

[bb3] Flack, H. D. (1983). *Acta Cryst.* A**39**, 876–881.

[bb4] Ou, G.-C., Zhang, M. & Yuan, X.-Y. (2008*a*). *Acta Cryst.* E**64**, m1010. 10.1107/S1600536808020564 PMC2961933 21203004

[bb5] Ou, G.-C., Zhang, M., Yuan, X.-Y. & Dai, Y.-Q. (2008*b*). *Acta Cryst.* E**64**, m1588. 10.1107/S1600536808038051 PMC2960142 21581188

[bb6] Sheldrick, G. M. (1996). *SADABS* University of Göttingen, Germany.

[bb7] Sheldrick, G. M. (2008). *Acta Cryst.* A**64**, 112–122. 10.1107/S0108767307043930 18156677

[bb8] Westrip, S. P. (2010). *J. Appl. Cryst.* **43**, 920–925.

